# Treating the Different Phenotypes of Behçet's Syndrome

**DOI:** 10.3389/fimmu.2019.02830

**Published:** 2019-12-06

**Authors:** Alessandra Bettiol, Gulen Hatemi, Lorenzo Vannozzi, Alessandro Barilaro, Domenico Prisco, Giacomo Emmi

**Affiliations:** ^1^Department of Experimental and Clinical Medicine, University of Florence, Florence, Italy; ^2^Department of Neurosciences, Psychology, Drug Research and Child Health (NEUROFARBA), University of Florence, Florence, Italy; ^3^Division of Rheumatology, Department of Internal Medicine, Istanbul University–Cerrahpaşa, Istanbul, Turkey; ^4^Eye Clinic, Careggi Teaching Hospital, University of Florence, Florence, Italy; ^5^Department of Neurology 2 and Multiple Sclerosis Regional Referral Centre, Careggi University Hospital, Florence, Italy

**Keywords:** Behçet's syndrome, phenotypes, cluster analysis, anti-TNF-α, DMARDs

## Abstract

Behçet's syndrome (BS) is a multisystemic vasculitis, characterized by different clinical involvements, including mucocutaneous, ocular, vascular, neurological, and gastrointestinal manifestations. Based on this heterogeneity, BS can be hardly considered as a single clinical entity. Growing evidence supports that, within BS, different phenotypes, characterized by clusters of co-existing involvements, can be distinguished. Namely, three major BS phenotypes have been reported: (a) the mucocutaneous and articular phenotype, (b) the extra-parenchymal neurological and peripheral vascular phenotype, and (c) the parenchymal neurological and ocular phenotype. To date, guidelines for the management of BS have been focused on the pharmacological treatment of each specific BS manifestation. However, tailoring the treatments on patient's specific phenotype, rather than on single disease manifestation, could represent a valid strategy for a personalized therapeutic approach to BS. In the present literature review, we summarize current evidence on the pharmacological treatments for the first-, second-, and third-line treatment of the major BS phenotypes.

## Introduction

Behçet's syndrome (BS) is a multisystemic vasculitis (1, 2), characterized by a broad spectrum of clinical involvements, including mucocutaneous, ocular, vascular, neurological, and gastrointestinal manifestations (1, 3). The different clinical manifestations may present alone, or co-exist in the same patient (4, 5). Cluster analyses and multivariate techniques have been applied to identify common clusters of BS manifestations, and, to date, three main disease phenotypes have been described: (a) the mucocutaneous and articular phenotype, (b) the extra-parenchymal neurological and peripheral vascular phenotype, and (c) the parenchymal neurological and ocular phenotype (Table 1).

**Table 1:** Major clusters of Behçet's manifestations and therapeutic options for the different disease phenotypes.

**Phenotypes**	**Evidence for the phenotype**	**Type of study; Cluster of manifestations**	**Treatment**	**Major evidence for the treatment**
Mucocutaneous and articular phenotype	Diri et al. (6)	Analysis of variance; Papulopustular lesions and arthritis	Colchicine (+/– steroids)	Clinical trials: (7–9)
	Tunc et al. (10)	Factor analysis; Genital ulcers, and erythema nodosum	Azathioprine	Clinical trial: (11)
	Hatemi et al. (12)	Analysis of variance; Enthesopathy, acne and arthritis	IFN α	Clinical trial: (13) Observational study: (14)
	Karaca et al. (15)	Factor analysis; Genital ulcers, and erythema nodosum with or without oral ulcers; papulopustular skin lesions and joint involvement with or without oral ulcers	Anti TNF-α	Clinical trial (for etanercept): (16). Observational studies and case series (for adalumumab and infliximab): (17, 18)
	Yazici et al. (4).		Anti Interleukin-1	Clinical trial: (19) Case series: (20)
	Kurosawa et al. (21)	Correspondence analysis; Onset age: 30–39 years, skin lesions, arthritis	Secukinumab	Case series: (22)
Extra-parenchymal neurological and peripheral vascular involvement phenotype	Tunc et al. (23)	Chi-square test; Cerebral venous sinus thrombosis and peripheral major vessel disease	Anticoagulant + immunosuppressant +/– steroids	Retrospective studies and case series (for anticoagulation): (24–26)
	Saadoun et al. (27)	Chi-square test; central nervous system involvement and extraneurologic vascular lesions		Retrospective studies (for immunosuppressants in general): (28–30) (for anti TNF-α): (31, 32)
	Tascilar et al. (33)	Correspondence analysis; Cerebral venous sinus thrombosis and pulmonary artery involvement		
	Shi et al. (24)	Chi-square test; extra cranial vascular involvement and cerebral venous sinus thrombosis.		
Parenchymal central nervous system and ocular phenotype	Bitik et al. (34)	Chi-square test; posterior uveitis and parenchymal neurological involvement	Steroid pulses	Clinical trial (for ocular involvement): (35)
	Kurosawa et al. (21)	Correspondence analysis; male, eye disease, HLA-B51 (+), neurologic involvement	Azathioprine	Clinical trial: (11, 36)Observational evidence (for azathioprine alone or in combination): (37, 38)
			Anti TNF-α	Interventional study (for infliximab): (39, 40) Observational studies (for infliximab): (41, 42) Clinical trials (for adalimumab): (43, 44) Observational studies (for adalimumab): (41, 45, 46)
			Cyclophosphamide	Observational study: (47–49)
			Tocilizumab	Observational study: (50) Case report/series: (51–53)

While extensive and updated literature reviews and recommendations exist for the treatment of the single BS involvements (6, 54), to date, poor attention has been given to the management of the different clusters of BS manifestations. The present review aims to provide clinicians evidence-based data to guide the choice of the most appropriate first-, second-, and third-line therapeutic approaches of the major BS phenotypes. Namely, first-line treatments should be considered as first options for naïve patients, based on current EULAR recommendations and on the extensive literature evidence on their efficacy (55). In patients intolerant or resistant to first-line drugs (or with severe BS forms), second or further lines of treatment should be considered, based on the availability of literature evidence to guide their use.

## Mucocutaneous and Articular Phenotype

### Evidence on the Phenotype

Skin-mucosa ulcerations are the most common, and usually the earliest, manifestations of BS, and recurrent oral and genital lesions are the hallmark of this syndrome (1). While one third of the BS population presents with only recurrent mucocutaneous symptoms (56, 57), a not negligible proportion of patients presents both mucocutaneous and articular involvements. The association between acne and arthritis has been demonstrated in past decades (6), but it is suggested that also enthesitis was part of this clinical association (4, 21).

Indeed, BS shares with seronegative spondyloarthritides (SpA) common pathogenetic mechanisms and genetic susceptibility, including the interleukin (IL)-23 and IL-17 pathways (1). Moreover, the involvement of major histocompatibility complex (MHC) class I alleles both in BS and in SpA [human leukocyte antigen (HLA)-B^*^51 and HLA-B^*^27, respectively] led to the unifying concept of “MHC-I-opathies” (58).

### First- and Second-Line Treatments

**Figure 1:**
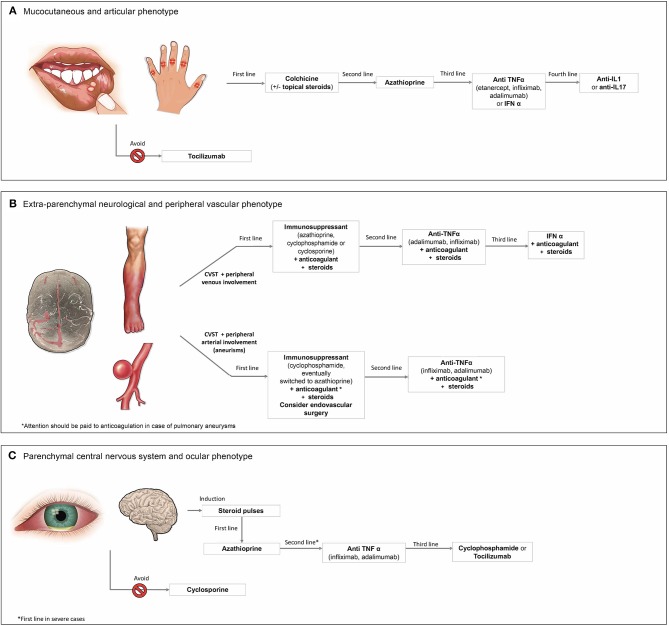
Therapeutic approach to the **(A)** mucocutaneous and articular phenotype, **(B)** extra-parenchymal neurological and peripheral vascular phenotype, and **(C)** parenchymal central nervous system and ocular phenotype of Behçet's syndrome.

In patients newly diagnosed with BS and presenting this “mucocutaneous and articular phenotype,” first-line treatment should be based on colchicine (Figure 1A). Colchicine has long been used in BS, with first evidence on its beneficial results for the treatment of erythema nodosum and arthralgia dating back to 1980 (7). Later on, two randomized controlled trials (RCTs) showed that colchicine led to a significant improvement of oral and genital ulcers, erythema nodosum, and articular symptoms (8, 9). The 2018 EULAR recommendations support the use of colchicine as first-line systemic treatment, especially when the dominant lesions are erythema nodosum or genital ulcers (55).

In patients intolerant or resistant to colchicine, azathioprine (AZA) can represent an effective second-line treatment. Efficacy of AZA for oral and genital ulcers and for arthritis was documented in a 2-year RCT of AZA (2.5 mg per kilogram of body weight per day) (11). In addition, AZA was superior to placebo in preventing new eye disease involvement (11). Based on this evidence, AZA can be considered as a first-line treatment in patient carrying also mild ocular involvement.

### Third Line Treatments

In patients inadequately controlled with, or intolerant to, the aforementioned synthetic immunosuppressive regimen, the use of biologic strategies, namely, with anti-TNF-α, or interferon (IFN) α should be considered. Among anti-TNF-α agents, only etanercept (ETN) 25 mg twice a week for 4 weeks has been studied in a trial on 40 BS patients with mucocutaneous disease and/or arthritis, showing a significant decrease of oral ulcers, nodular, and papulopustular lesions (16). However, data on the efficacy of ETN on arthritis were not conclusive, and the effects of this drug on genital ulcers were comparable with those in the placebo group. Conversely, the use of adalimumab (ADA) and infliximab (IFX) is supported by different observational studies and case series (17). Among them, a multicenter study on 124 BS patients showed that the clinical response to the treatment with either ADA or IFX was 88% for mucocutaneous involvement and 77.8% for articular involvement (18).

The efficacy of IFN α in the “mucocutaneous and articular phenotype” was reported in a retrospective observational study on 18 BS patients, treated for 12 weeks (14). Later on, in an RCT, IFN α was shown to control oral and genital ulcers, papulopustular lesions, erythema nodosum-like manifestations, and articular symptoms, while improving the severity and the frequency of ocular attacks (13). Of note, the safety profile of this drug deserves some attention, since adverse events including flu-like syndrome, leukopenia, transient elevation of liver enzymes, as well as psychiatric disorders have been reported (13). Bone marrow suppression may be even more pronounced when used together with AZA (37).

### Fourth-Line Treatments

In patients resistant, refractory, or intolerant to anti-TNF-α agents or IFN α, evidence supports the use of other biologic treatments for this phenotype. Specifically, some evidence (although not consistent) supports the use of IL-1 inhibitors anakinra (ANA) or canakinumab (CANA) (19, 20, 59). Specifically, in an adaptive, two-phase pilot open label study conducted on six BS patients with active mucocutaneous manifestations and with concomitant arthritis, ANA at an optimal dose of 200 mg daily provided partial control of resistant mucocutaneous and articular involvements (19).

In a recent case series of five BS patients with active and refractory mucocutaneous and articular manifestations, the anti-IL17 agent secukinumab (either 150 mg and 300 mg/month) was associated with a consistent improvement of both mucocutaneous and articular involvements (22).

Regarding other promising treatments, growing evidence supports the use of ustekinumab (60–62) and apremilast (63, 64) for the control of mucocutaneous involvements. Of note, following a phase 2, placebo-controlled trial and a phase 3, multicenter, placebo-controlled study on 207 patients with active BS (64, 65), apremilast is the only drug currently approved by the Food and Drug Administration (FDA) for the treatment of mucocutaneous manifestations in BS. However, as no clear evidence exists on the efficacy of apremilast for the control of articular BS involvement, the role of this drug for the management of the mucocutaneous and articular BS phenotype is yet unclear.

On the other hand, the use of the anti-IL6R tocilizumab (TCZ) should be avoided in patients presenting this phenotype, considering that TCZ-induced exacerbation of mucosal ulcers has been reported (66, 67).

## Extra-Parenchymal Neurological and Peripheral Vascular Phenotype

### Evidence on the Phenotype

Superficial venous thrombosis (SVT) and deep vein thrombosis (DVT) are the most frequent vascular manifestations of BS, affecting altogether up to 40% of patients (31, 68–70). DVT mainly involves the inferior, but also the superior limbs, while venous thrombosis of atypical locations (TAL) have been described (31, 69–71). At the cerebral level, non-parenchymal vascular central nervous system (CNS) involvements include cerebral venous sinus thrombosis (CVST), arterial occlusion, and/or aneurysms (72). CVST represents 10–30% of all neurological BS manifestations (73). The concomitant presence of both cerebral arterial manifestations and CVST is extremely rare (74). In an analysis of 88 patients with CNS disease, a significant association was found between peripheral vascular disease and extra-parenchymal CNS involvement (i.e., dural sinus thrombi), while a poor association was found between parenchymal neurological and peripheral vascular involvements (23). In a retrospective study involving 21 BS patients with CVST, the presence of extra cranial thrombosis was documented in 52% of patients (24). In a cohort study on 820 patients, CVST was reported in 64 cases. Among them, the presence of concomitant extra-neurological vascular lesions was significantly more frequent than in patients without CVST (27).

The concomitant presence of central and peripheral vascular involvements is probably sustained by common thrombogenic mechanisms. Namely, inflammation-induced thrombosis has been described in BS, with neutrophils playing a critical role in promoting oxidative stress, inflammation, and consequent endothelial dysfunctions (31, 75, 76). In this context, immunosuppression represents a key strategy for the therapeutic management of central and peripheral vascular involvements (31, 71).

### CVST and Peripheral Venous Involvements

#### First-Line Treatments

High-dose glucocorticoids are the mainstay treatment for rapid induction of remission in CVST (60). There is no consensus on the use of additional anticoagulants or immunosuppressants, since recurrence is infrequent with this manifestation. In a recent literature review (31), we reported that anticoagulation has a predominant role in the management of BS-related CVST (24, 25, 31, 77), while it is yet unclear if the use of concomitant immunosuppressants influences the risk of sequalae or relapses (24). A recent case series of 7 patients with BS-associated CVST suggested that anticoagulant therapy might be safely discontinued during follow-up, in the presence of optimal BS therapeutic management with steroids alone or in combination with immunosuppressive drugs (26). On the other hand, the use of immunosuppressants is pivotal in the control of DVT and SVT (28–31), while concomitant use of anticoagulants in these peripheral associations has been associated with controversial benefits (31), except for preventing the occurrence of severe post-thrombotic syndrome (78).

Thus, the first-line treatment of patients carrying the “extra-parenchymal neurological and peripheral vascular phenotype” should be based on immunosuppressants with the addition of anticoagulants in selected patients (Figure 1B). Specifically, in CVST associated with SVT and/or DVT, evidence suggests as first-line treatment AZA, cyclophosphamide (CYC) or cyclosporine (CSA) (31, 66).

#### Second- and Third-Line Treatments

In patients with refractory peripheral venous thrombosis, anti-TNF-α, namely, ADA, or IFX, should be used, alone or in combination with traditional disease-modifying anti-rheumatic drugs (DMARDs) (1, 31).

Eventually, IFN α can be considered a therapeutic approach in selected cases (79). In a prospective study on patients with lower-extremity DVT, the treatment with IFN α accounted for a good recanalization and low relapse rates (80). According to the current EULAR recommendations, the treatment with IFN α can be considered in selected cases (55). However, the role of this treatment for the control of CNS vascular involvements is still unclear.

### CVST and Arterial Involvements

#### First-Line Treatments

First-line treatment of patients carrying the CVST and peripheral arterial involvements should be based on immunosuppressants, mainly CYC, in association with high-dose steroid and (after excluding pulmonary aneurysms) with anticoagulants in selected patients (55). According to the last EULAR recommendations, CYC can be administered as monthly intravenous pulses, while glucocorticoids are given as three intravenous methylprednisolone pulses followed by oral prednisolone (or prednisone) at the dose of 1 mg/kg/day (55) (Figure 1B). For the maintenance treatment, CYC can be replaced by AZA (1).

Notably, sometimes peripheral aneurysms require emergency surgery or stenting (55). The use of prednisone alone or in combination with AZA is recommended also in patients with pseudoaneurysm, before endovascular treatment (81, 82), while in the days after surgery, successful use of hydrocortisone plus CSA has been reported (81).

#### Second-Line Treatments

In patients with arterial involvements refractory to conventional DMARDs, second-line treatment with anti-TNF-α (namely IFX or ADA) should be considered (32, 55). In an observational study on 13 BS patients with refractory pulmonary artery involvement, anti-TNF-α effectively controlled these involvements, although it did not prevent their development (32).

An effective use of ADA following unsuccessful treatment with prednisone, CYC, and conventional immunosuppression was reported also in a patient with right ventricular thrombus and large aneurysms of the pulmonary arteries leading to recurrent episodes of hemoptysis (83), as well as in a case of life threatening bilateral pulmonary artery aneurysms and thrombotic disease (84).

## Parenchymal CNS and Ocular Phenotype

### Evidence on the Phenotype

The involvement of the parenchymal CNS is a major cause of morbidity and mortality in BS (73, 85). In a study conducted on 200 neuro-BS out-patients, 162 had parenchymal CNS involvement (72). In a first post-mortem study on a BS patient with parenchymal involvement, a cell infiltration was found around the central retinal artery within the optic nerve (86). Eye involvement is present in around half of BS patients, with a higher prevalence in males, and a lower prevalence among elderly (87). Ocular involvement is one of the most disabling complication in BS (87). In a retrospective observational study on 295 BS patients, a significant association between posterior uveitis and parenchymal CNS involvement was reported (34). Furthermore, male sex, eye disease, HLA-B51 positivity, and neurologic involvement are features identifying a specific cluster of BS patients (21).

Of note, in a recent study on 30 BS patients with ocular involvement without overt neurological symptoms, silent neurologic manifestations, including neuropsychological deficits, subcortical magnetic resonance imaging (MRI) lesions, and non-structural headache, were found in a relevant proportion of patients (88).

Although the pathogenetic mechanisms sustaining the concomitant occurrence of ocular and neurological BS involvements have never been described, the embryogenic process and the involvement of the neural tube and neural crest in the organogenesis of the eye might account for this association (89).

### First-Line Treatments

No RCT has determined the optimal therapeutic management of neurological BS, nor for its association with ocular involvement (90). The induction treatment of acute severe neuro-BS is mainly based on high-dose corticosteroids, followed by the gradual tapering of the oral doses over 3–6 months (90–92) (Figure 1C). As first-line treatment for the “parenchymal neurological and ocular phenotype,” AZA should be used (90). Specifically, according to current EULAR recommendations, AZA at the dosage of 2.5 mg/kg per day is recommended as first-line immunosuppressive agent for both ocular and parenchymal manifestations (1, 55). In case of severe ocular and parenchymal CNS involvements, the use of second-line options, namely, anti-TNF-α drugs, should be considered as first-line treatment.

### Second-Line Treatments

In refractory cases, the use of anti-TNF-α can be considered (54). Indeed, consistent observational evidence supports the use of IFX (at the dose of 5 mg/kg) in both neurological and ocular BS involvements (1, 39, 55).

ADA at the dose of 40 mg every other week represents a valid second-line alternative (1). Effective use of ADA for non-infectious uveitis was first reported in two RCTs on few BS patients (43, 44, 93). Later observational evidence confirmed the benefits of this treatment in BS-related uveitis. In four Italian multicenter observational studies, treatment with either ADA or IFX proved effective for the treatment of refractory retinal vasculitis (45, 94–96). In another recent observational study on 106 patients with uveitis, ADA was associated with high rates of ocular control, effective steroid tapering, and good preservation of visual acuity, also in the absence of concomitant DMARDs treatment (46). Similarly, increasing observational evidence supports the use of ADA or IFX in neuro-BS (41).

### Third-Line Treatments

Further therapeutic options for this phenotype are CYC or TZC. According to a 10-year longitudinal study, CYC (1 g/month for 6 months and then every 2–3 months), in association with AZA and prednisolone, was the best treatment for retinal vasculitis, before opting for biologic agents (47). Nevertheless, in a single masked trial (97), CYC was found to be inferior to CSA in controlling ocular involvements; however, CSA cannot be considered as a valid approach for this phenotype, as it is contraindicated in active neuro-BS.

CYC (1 g/month for 6–12 months or 0.8 g/m^2^) has been associated also with some benefits in parenchymal neuro-BS (79, 98). In a French study on 115 patients with parenchymal neuro-BS, the use of CYC (*n* = 53) resulted as effective as AZA (*n* = 40) and steroids alone (*n* = 19) in preventing relapses (48). Furthermore, in patients with moderate to severe disability (i.e., with moderate to severe disability scoring 3 or more in the modified Rankin scale for the assessment of the disability), CYC was associated with slightly higher event-free survival rates at 1 to 10 years as compared to AZA, although without statistical significance. In a Korean study on 22 patients with parenchymal neuro-BS, a treatment with CYC associated with steroids was found to be as effective as treatment with steroids alone in preventing relapses (49).

The anti-IL6R TCZ is a promising treatment in the “parenchymal neurological and ocular phenotype.” Results from case reports and case series suggest its effectiveness for refractory neuro-BS (51–53), while a recent retrospective study on 11 patients with refractory uveitis reported rapid and sustained ocular improvement in all the patients (50). However, the use in daily clinical practice of TZC for treating this phenotype still needs more studies for further confirmation. As for other non-biologic alternatives, IFN α is highly effective for ocular control (55), and might have a potential role also for refractory neuro-BS (99, 100). Notably, the use of CSA should be avoided in the “parenchymal neurological and ocular phenotype” (55). In fact, while effective in ocular manifestations, an increased risk of CNS manifestations in patients taking this drug has been reported (101–103).

## Conclusions

Growing evidence supports that, within the definition of BS, different clinical phenotypes can be distinguished. Thus, therapeutic strategies could be tailored on patient's specific phenotype, rather than on single disease manifestations.

Based on available literature, patients carrying the “mucocutaneous and articular” BS phenotype should start a first-line treatment with colchicine, alone or in combination with corticosteroids, while AZA can be considered in patients resistant or intolerant to colchicine. The use of anti-TNF-α or IFN α should be reserved to truly refractory or severe forms.

In patients presenting the “extra-parenchymal and peripheral vascular phenotype,” use of immunosuppressants and additional anticoagulants in selected patients should be recommended. Traditional immunosuppressants (mainly AZA) should be started as first-line treatment, while anti-TNF-α agents represent a valid second-line treatment. IFN α may be a promising alternative.

As for the “parenchymal neurological and ocular phenotype,” first-line treatment with AZA is recommended after an induction therapy with high-dose steroids. In patients with a severe presentation, or those who are intolerant or refractory to AZA, anti-TNF-α drugs should be used.

However, comparative studies should be performed to evaluate whether this phenotype-based therapeutic approach is associated with a better effectiveness as compared to the classic organ-based approach.

## Author Contributions

GE and ABe conceived the work. ABe performed the literature review, assisted by GE and GH. ABe and GE drafted the paper. GH, LV, ABa, and DP critically revised and implemented the manuscript. All authors approved the final version of this manuscript.

### Conflict of Interest

The authors declare that the research was conducted in the absence of any commercial or financial relationships that could be construed as a potential conflict of interest.

## Abbreviations

ADA: adalimumab
ANA: anakinra
AZA: azathioprine
BS: Behçet's syndrome
CANA: canakinumab
CNS: central nervous system
CSA: cyclosporine
CVST: cerebral venous sinus thrombosis
CYC: cyclophosphamide
DMARDs: disease modifying anti-rheumatic drugs
DVT: deep vein thrombosis
ETN: etanercept
HLA: human leukocyte antigen
IFN: interferon
IFX: infliximab
IL: interleukin
MHC: major histocompatibility complex
RCT: randomized controlled trial
SpA: spondyloarthritides
SVT: superficial venous thrombosis
TAL: thrombosis of atypical locations
TCZ: tocilizumab.
